# Implantable strain sensor to monitor fracture healing with standard radiography

**DOI:** 10.1038/s41598-017-01009-7

**Published:** 2017-05-04

**Authors:** Hunter Pelham, Donald Benza, Paul W. Millhouse, Nathan Carrington, Md. Arifuzzaman, Caleb J. Behrend, Jeffrey N. Anker, John D. DesJardins

**Affiliations:** 1 Department of Bioengineering, Clemson University, Clemson, SC USA; 2 Department of Chemistry, Clemson University, Clemson, SC USA; 3 Department of Mechanical Engineering, Clemson University, Clemson, SC USA; 4 Department of Electrical Engineering, Clemson University, Clemson, SC USA; 5 Virginia Tech Carilion School of Medicine and Research Institute, Roanoke, VA USA

## Abstract

Current orthopaedic clinical methods do not provide an objective measure of fracture healing or weight bearing for lower extremity fractures. The following report describes a novel approach involving *in-situ* strain sensors to objectively measure fracture healing. The sensor uses a cantilevered indicator pin that responds to plate bending and an internal scale to demonstrate changes in the pin position on plain film radiographs. The long lever arm amplifies pin movement compared to interfragmentary motion, and the scale enables more accurate measurement of position changes. Testing with a human cadaver comminuted metaphyseal tibia fracture specimen demonstrated over 2.25 mm of reproducible sensor displacement on radiographs with as little as 100 N of axial compressive loading. Finite element simulations determined that pin displacement decreases as the fracture callus stiffens and that pin motion is linearly related to the strain in the callus. These results indicate that an implanted strain sensor is an effective tool to help assess bone healing after internal fixation and could provide an objective clinical measure for return to weight bearing.

## Introduction

While a variety of fracture stabilization therapies are available to surgeons for internal fixation, the inability to directly evaluate healing remains a limiting factor when assessing patient recovery and return to a pre-injury activity level. Tibial fractures are the most common long-bone fracture (36.7% of all long-bone fractures in adults) and also happen to be the most common site of long-bone nonunion^1^. Patients with nonunion are more likely to have additional fractures during follow-up, to require various types of in-patient and surgical care (including amputation), to be prescribed pain medications (especially strong opioids), and to use more outpatient physical therapy than those with proper bony union^2^. Median total costs of care for patients with nonunion were found to be more than double those without ($25,556 vs. $11,686 according to 2006 data)^2^. To help prevent complications such as non-union or malunion, refracture and implant failure, physicians often limit weight bearing for an extended time, often 12 weeks or longer, to allow for adequate bone growth^3^. While premature weight bearing can increase the rate of complications, unnecessarily delaying weight bearing results in productivity loss with indirect costs from lost wages and places additional burdens on the healthcare system. Identification of post-operative complications (non-union, infection, implant loosening, etc.) throughout the recovery process, while critical to effective treatment, is often difficult with existing internal fixation methods.

Current clinical methods of monitoring bone healing and identifying complications following an open-reduction and internal fixation (ORIF) are typically limited to a combination of symptomatic, physical examination, and radiographic findings. Interpretation is subjective, influenced both by the patient’s candid description of symptoms and by the treating physician’s clinical judgment^4^. Standard radiographic assessment lacks the sensitivity to detect small amounts of fracture movement to directly measure fracture healing^5^. For example, to assess whether spinal fusion had occurred, Song *et al*. measured the displacement of spinous processes under flexion and extension; they found a 95% limit of inter-observer difference ranging from −1.6 mm to 1.3 mm^6^. For long bones essentially only the size of the callus can be quantified on standard radiographs, which does not correlate with stiffness^7^. Physicians can utilize three-dimensional CT scanning to more closely monitor bone density and the achievement of bony union. However, CT scans are expensive and typically expose patients to about 300 fold more radiation than plain film radiographs. Despite its prevalence, traditional radiographic assessment has thus proven unreliable as a tool for monitoring the fracture healing process^8^.

The low sensitivity of conventional X-rays can be improved by differentially loaded radiostereometric analysis which monitors the motion of radiopaque spherical beads implanted in the tissue or bone by simultaneously acquiring two images at different angles. Prior studies have shown that the injection of several tantalum beads near the fracture site can be tracked with up to 20–50 µm of resolution. Fracture stiffness and healing can then be monitored by bead motion under load^9^. However the clinical utility of this method is offset by the specialized instrumentation and training requirements, the increased cost and time of injecting beads during surgery, and complications from bead placement and migration, all of which limit widespread adoption. There is a need for new objective methods of monitoring fracture motion and bone healing in internal fixation applications that are easily implemented into standard clinical practice with minimal additional equipment, cost, or complexity.

One measure of fracture healing and bone union is fracture stiffness. Indirect measurements of fracture stiffness are possible by monitoring the mechanical response of fixation devices since load transfers from the fixator to the callus as the bone heals. Previous studies have successfully quantified bone healing patterns by monitoring strain on external fixators^10, 11^. Richardson *et al*. found that a fracture stiffness of 15 Nm/degree was an accurate threshold to indicate full weight bearing capability through indirect stiffness measurements^12^. Applying this value in clinical trials resulted in safe implant removal 2.3 weeks earlier than usual and a reduction in re-fracture complications from 7% (n = 117) to 0% (n = 95) of patients^12^. However, these methods and improvements are limited to external fixation applications, or complex *in-vivo* instrumented implants. It is impractical to manage fractures using an external fixator and these are typically used only temporarily in the in-patient setting prior to ORIF. The majority of fractures are treated with internal fixation for which there is no validated or widely accepted method to objectively monitor bone healing non-invasively.

**Figure 1:**
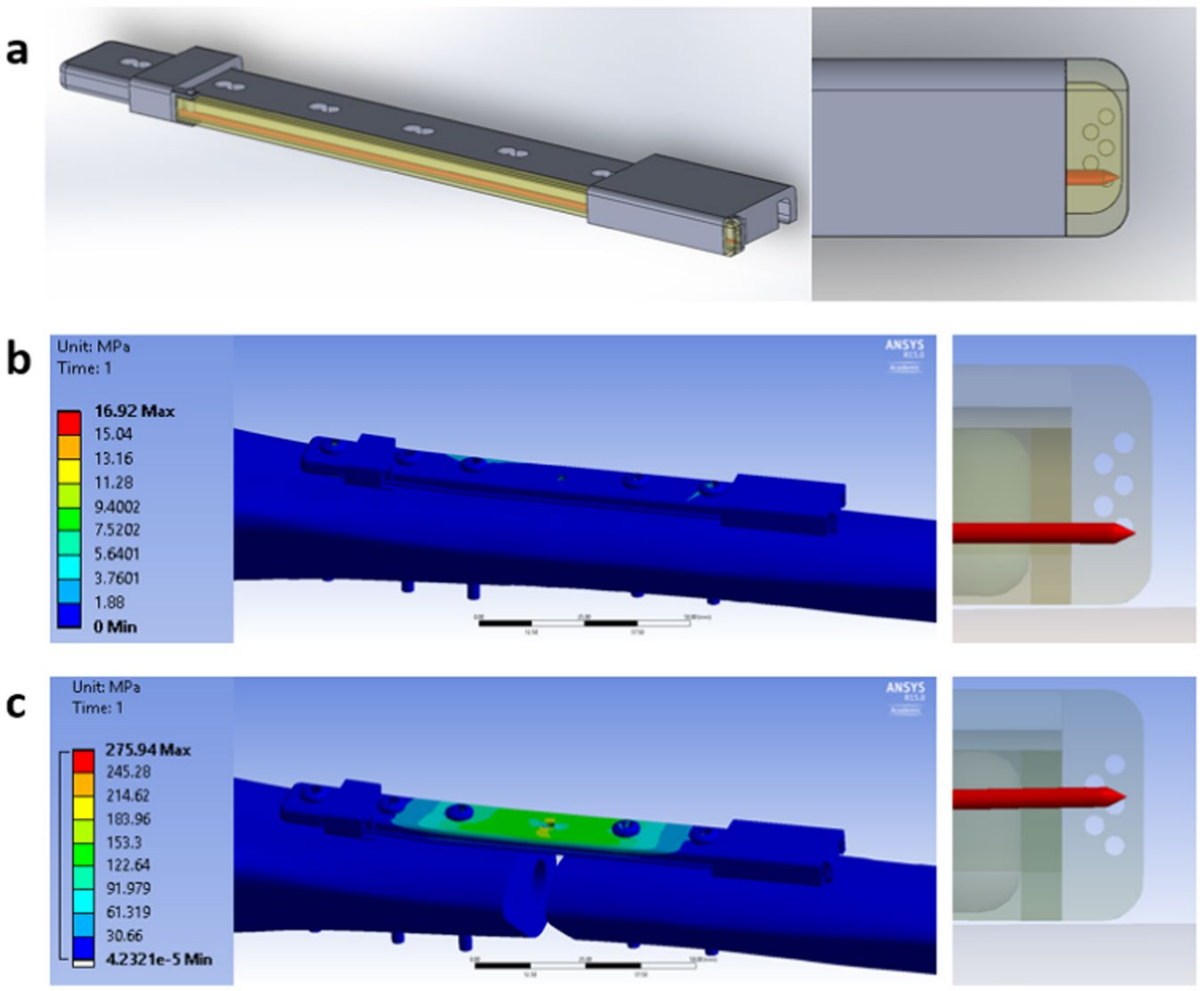
Passive strain sensor design. (**a**) Solidworks model of passive strain sensor components mounted to an orthopedic plate modelled after Synthes 4.5 mm proximal tibia plate. (**b**) FEA of intact tibia geometry with attached plate sensor. Note no change in sensor response when comparing no load to 400 N compressive load. (**c**) FEA of tibia with 1 cm osteotomy treated with internal fixation. The plate is supporting all of the axial load, resulting in plate bending and pin displacement. Indicator pin rests between holes 3 and 4 with a 400 N axial compressive load.

We propose an implantable strain sensor that easily mounts to existing internal fixation orthopedic plates. The sensor (Fig. 1a) consists of a cantilevered radiopaque indicator pin that spans the fracture and an internal scale to allow for easy tracking of pin displacement with standard radiography. The cantilevered pin provides a degree of mechanical gain based on the ratio of pin length to bone diameter. This mechanical gain is advantageous since angular deflection and interfragmentary motion are normally too small to be effective indicators of bone healing with standard radiography^13^. As the plate bends from load sharing with the healing bone, the pin moves relative to the hole pattern on the internal scale. The initial design features five 0.5 mm diameter holes that are clearly visible with radiography unless obstructed by the indicator pin. This design is capable of 250 µm resolution compared to the 2–5 mm resolution with conventional x-ray assessment when measuring interfragmentary displacement^14^. The combination of this mechanical gain and the resolution when reading the internal scale allows the sensor to detect and measure strain in clinically relevant ranges more effectively than current methods.

The sensor is quickly secured to the orthopedic plate by tightening a pair of set screws. Once installed, this provides unambiguous access to the mechanical response of the orthopedic plate and thereby the fracture callus stiffness during all stages of bone healing. Acquiring readings of the sensor response is obtained simply through plain radiography already utilized in most clinics throughout follow-up. This degree of implant monitoring is currently unavailable for internal fixation applications and can increase a surgeon’s ability to identify complications that may be missed with traditional methods. In addition, delayed weight bearing can be avoided since protracted estimations of fracture healing would be alleviated. On the other hand, weight bearing may be delayed if necessary based on serial stiffness readings, thus providing an additional security measure for the patient and physician.

## Results

### Finite element analysis (FEA)

FEA was used to study fracture strain and the response of the prototype strain sensor under a variety of loading conditions and fracture properties. A 400 N axial compressive load was applied to the proximal end of an intact tibia (approximately one-half body weight for an 80 kg patient). These conditions resulted in no significant displacement of the indicator pin, as shown in Fig. 1b. A 10 mm osteotomy in the mid-section of the bone was then introduced (a common model for an unstable comminuted fracture) and the same 400 N compressive load was applied. An indicator pin displacement of 1.45 mm (sensor reading between holes 3 and 4) relative to the internal scale was observed, as shown in Fig. 1c. Once the 10 mm osteotomy is introduced, the plate supports the entire load across the fracture. The fully supported compressive load results in plate bending since the plate is positioned offset from the axis of the bone. The strain sensor responds to the plate bending by an increase in pin displacement relative to the internal scale.

**Figure 2:**
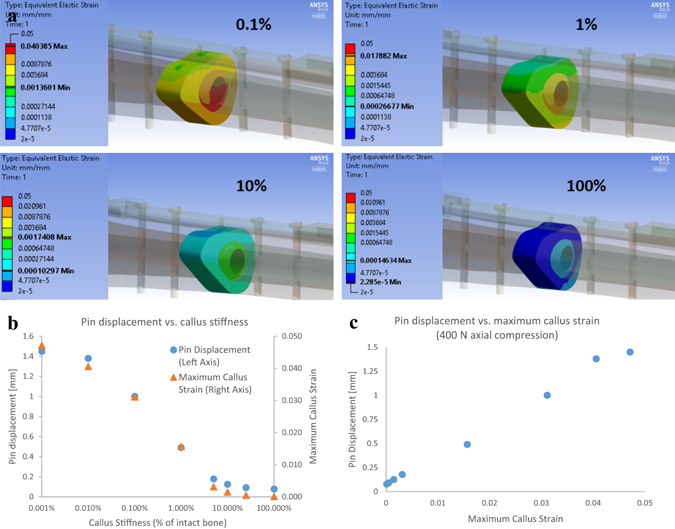
Fracture Callus FEA. (**a**) Strain distribution within the fracture callus for 400 N axial compression load with varying callus stiffness as a percent of cortical bone. (**b**) Pin displacement and maximum principal callus strain vs. callus stiffness. (**c**) Linear relationship between pin disp. and maximum callus principal strain.

The effects from replacing the fracture geometry with callus of varying stiffness was also studied. Prior studies have shown that as fracture callus matures during early healing, its stiffness exponentially increases from a Young’s modulus of ~5 × 10^4^ Pa for granulation tissue to ~2 × 10^10^ Pa for mature cortical bone, and that the ultimate strength of the callus also increases at approximately half the rate of the stiffness^15, 16^. In order to simulate this process in an FEA model, the callus stiffness was varied from 0.001–100% that of intact bone. The indicator pin displacement and maximum callus principal strain versus callus stiffness were then examined. Figure 2b shows a linear increase in pin displacement with maximum callus principal strain (R^2^ = 0.995). Note that approximately 1.5% callus principal strain causes nearly 0.5 mm of relative pin displacement, which is easily measured with the prototype sensor. Up to 2% interfragmentary strain is conducive to primary bone healing^17^. Figure 2c shows the relationship between pin displacement and callus stiffness. The FEA model demonstrates that as the callus stiffens, the orthopedic plate shares less of the load and its displacement decreases. Eventually the callus stiffness is high enough to offload the orthopedic plate, resulting in significantly reduced plate bending and sensor response.

### Orthopedic model

**Figure 3:**
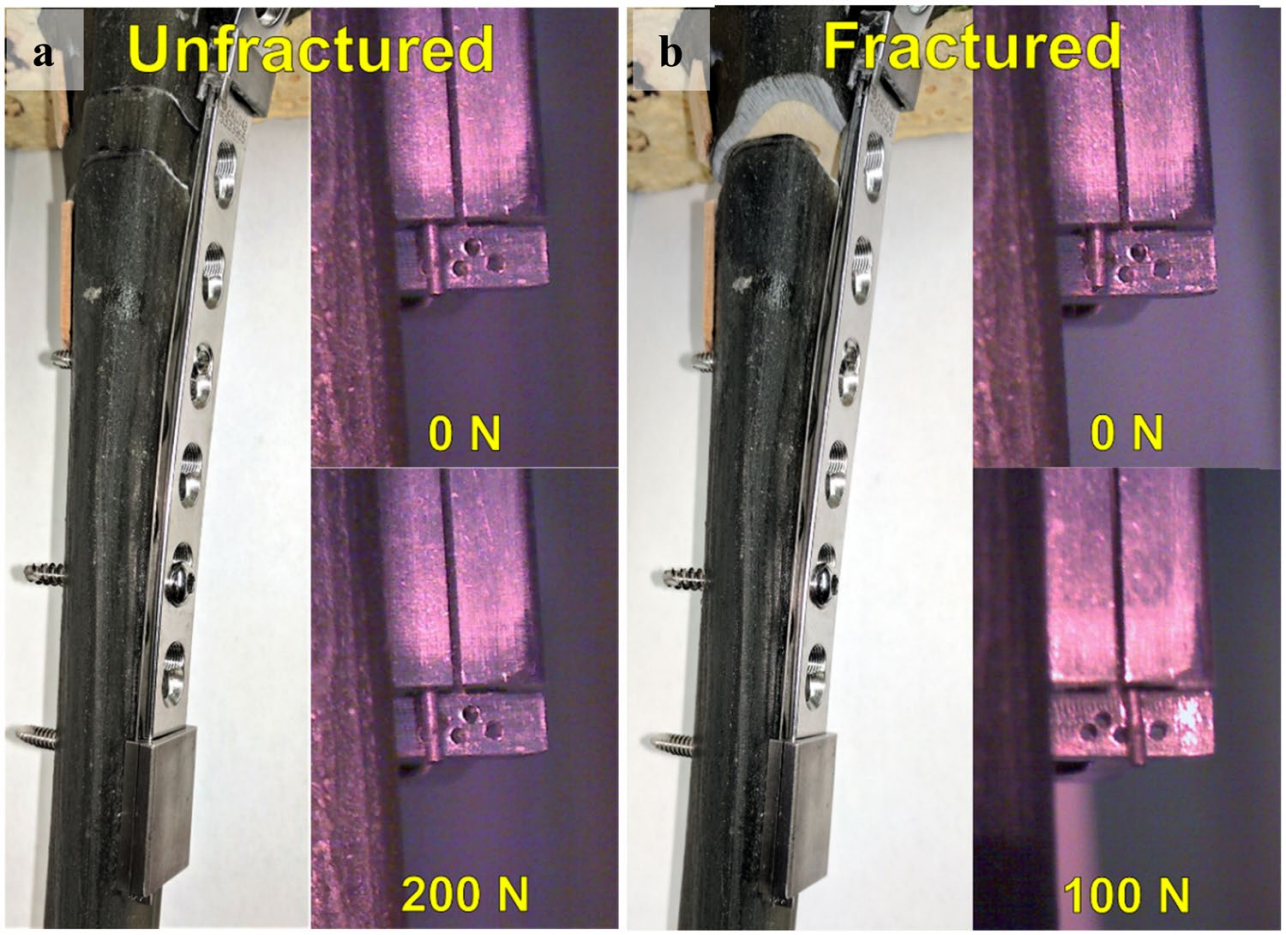
(**a**) Unfractured Sawbones^®^ model. (**b**) Fractured Sawbones^®^ model.

**Figure 4:**
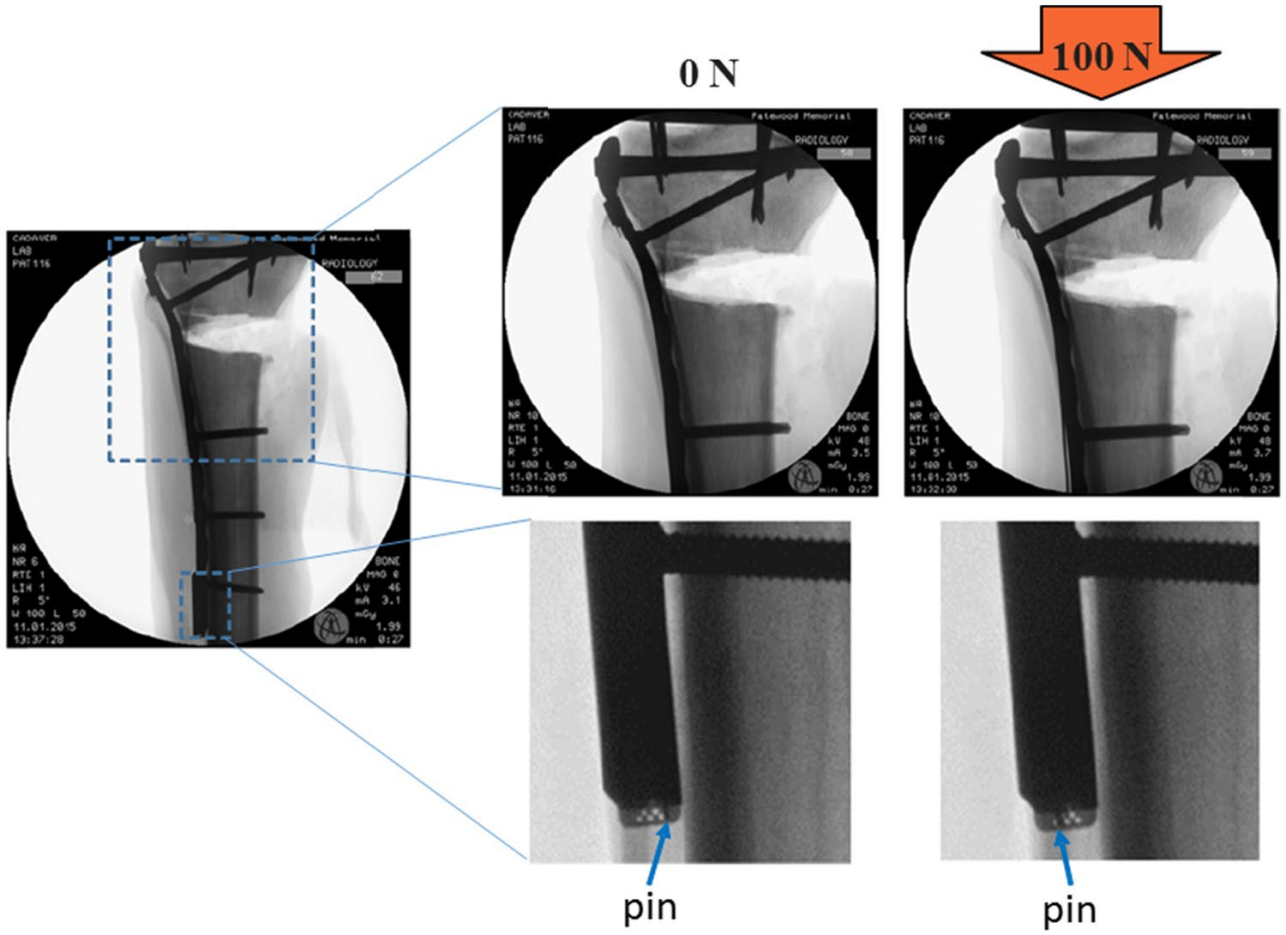
Radiographic images of the Sensor. The implant, fracture gap, and passive strain sensor are shown for both unloaded and 100 N compression loaded cases in a human cadaver tibia. The indicator pin moves from below hole 1 to covering hole 4 under the 100 N load.

After promising initial results from the FEA model, a prototype device was fabricated and fashioned to a Sawbones^®^ (Pacific Research Laboratories, Inc., Vashon Island, WA) tibial model. Specifically, an internal fixation plate-mounted strain sensor prototype was developed to monitor plate bending under load (Figs 3 and 4). Results of the experiments are presented below for the unfractured (Fig. 3a) and fractured (Fig. 3b) Sawbones^®^ tibia conditions. The indicator pin did not move perceptibly with the unfractured model under up to 200 N of loading; higher loading conditions were not attempted. In the fractured condition, the pin moved approximately three holes (1.5 mm) with 100 N of force applied. This response is larger than the FEA model (1.45 mm with 400 N load) partly because the fracture position is midshaft in the FEA analysis and more proximal in the Sawbones^®^ study; since most of the bending occurs at the fracture gap, the pin displacement is influenced by the bend angle and distance between the gap and distal end of the plate where the pin is read. Furthermore, implant stiffness can be controlled by the configuration of the orthopedic screws that fix the plate to the bone^18^. Specifically, implant stiffness decreases as bridging length of the screws increases^18^. Both cadaveric and Sawbones^®^ experiments featured implants with a greater distance between the fracture gap and the pin tip, as well as greater bridging length between orthopedic screws when compared to the FEA model. The combination of these factors accounts for the discrepancy in sensor response for a given load between the models. However, the displacement with the sawbones agreed reasonably well with the cadaveric experiment described below (2.25 mm displacement under 100 N).

### Cadaveric sensor response

**Figure 5:**
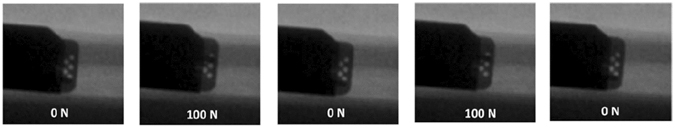
Radiographic images of sensor response over multiple cycles.

During human cadaveric testing, an intact bone loaded in compression resulted in no discernable change in the position of the indicator pin with standard radiography. However, once an unstable fracture was introduced to the tibia, steady movement of the indicator pin relative to the internal scale was clearly observed on radiographs as the compressive loading increased. The indicator pin moved from the first hole on the internal scale to the final hole at nearly 200 N. The indicator also successfully returned to the starting position when the compressive load was removed in each of the loading cycles. The sensor is easily read compared to radiographic images of the fracture gap when attempting to evaluate implant bending. Radiography of the sensor response and the fracture gap are presented in Fig. 4. The results were highly reproducible under cyclic loading, as shown in Fig. 5.

Through cadaveric testing and radiography it was demonstrated that the prototype strain sensor was capable of showing one of eleven distinct positions. Five potential readings resulted from the indicator pin covering one of the scale holes completely. Another five potential readings occurred when the indicator pin rested between two holes, partially covering both. The last potential reading was when the indicator pin rested below the first hole in the starting position.

## Discussion

It was determined that the prototype sensor can be read clearly through radiography images during human cadaver testing. The images of the sensor moving against a scale provided quantitative readings of plate bending and interfragmentary displacement with a resolution of half of a hole (250 µm) or better, compared to 2–5 mm resolution using traditional X-ray assessment of the fracture site. In addition, the device provided a mechanical gain factor compared to the intrafagmentary motion at the location of largest motion. Assuming that most of the plate bending occurs near the fracture (e.g., Fig. 2), and the bone is essentially rigid except within the fracture callus, the gain factor is approximately equal to the ratio of the length of the indicator pin past the fracture to bone diameter (a gain factor of around four for a 12 cm pin length on a 3 cm bone diameter). The mechanical gain of the system can be further improved with increased pin length or the incorporation of gears, pivots, levers, hydraulic actuators, or other mechanisms found in similar applications. One example of other mechanical gain mechanisms is a common machinist’s dial indicator used for measuring micrometer to millimeter displacements (in order to center work pieces in lathes, for example).

The sensor successfully tracked plate bending and interfragmentary displacement with both increasing and decreasing axial loading of the tibia with adequate repeatability. The position of the indicator pin reached its limit at just under 200 N of axial loading during human cadaver testing. This response was to be expected as the orthopedic plate supports all of the load at this point due to the absence of a fracture callus in early healing; as such, a patient should be instructed to not bear weight at this stage. As the callus forms and increases in stiffness, it begins to share load with the orthopedic plate. Load sharing between the bone and the plate shifts more to the callus as the bone heals, resulting in less plate bending and reduced sensor response for a given load^19, 20^. Eventually the callus becomes stiff enough to support enough load from the orthopedic plate to allow for safe weight bearing without the risk of implant fatigue failure. FEA results comparing changes in callus stiffness to sensor response are consistent with this process, and show the callus supporting a majority of the load at high callus stiffness values.

It is important to note that sensor response may be influenced by a number of factors, including plate material, screw stiffness, screw fixation, fracture gap size, plate position relative to the fracture gap and the orientation between the implant and any applied loads. Orthopedic plates composed of stiffer materials will bend less under a given load and result in smaller pin displacements. Plate curvature should not influence sensor performance as the sensor tracks changes in plate bending over time by comparing images taken at various stages of bone healing. The indicator pin is also formed to match the contour of the plate prior to initial mechanical loading so surrounding tissue does not disrupt pin motion. Small fracture gaps can partially close when the limb is compressed, resulting in less plate bending and pin displacement. Gap opening/closing under smaller bending loads could also be studied as well as other bending, shear, and twisting loads. The FEA presented in this paper explores one case of internal fixation with axial compression loading. The FEA is limited by this single configuration of fracture geometry, location, and implant positioning. The orthopedic model and cadaver experiments explored other configurations of plate geometry and fracture location with similar results, demonstrating generalizability of the concept. Comparisons of the computational, Sawbones^®^, and cadaveric models are limited by variations in bone and implant geometry between these studies. In addition, the cadaveric study did not explore variations in bone geometry between patients since only one cadaver tibia was mechanically tested. Additional studies exploring the factors that influence plate bending and sensor performance with multiple donor samples are recommended to further understand the capabilities of the device. Future simulations should explore a variety of fracture geometries and locations, as well as a range of implant orientations based on existing clinical practice.

The sensor does not require any specialized training or techniques to be read using traditional radiographic imaging. However parallax is a potential concern, wherein observing the sensor from different angles causes apparent migration of the pin relative to the hole. Specifically, parallax error is given by equation (1).

1 $${\rm{\Delta }}x={\rm{s}}\ast ({\tan }^{-1}\,{\theta }_{1}-{\tan }^{-1}\,{\theta }_{2})$$

where *s* is the separation between the pin and the hole, *θ*_1_ is the angle difference between the X-ray principle axis and the plate to normal for the X-ray taken with no load, and *θ*_2_ is the angle difference under load. For an extreme example, assuming that the separation between the pin and hole is 250 µm (half of a hole), *θ*_1_ is zero degrees, and *θ*_2_ is 30° (a very large misalignment requiring the patient to rotate their leg between X-ray images), parallax error would be 120 µm, only about a quarter of a hole which would not significantly affect readings. In practice, such large changes in X-ray acquisition angle would be obvious from the shape of the holes in the image (which become elliptical at large angles). Such large misalignment could trigger either reimaging the patient or more sophisticated analysis to account for the parallax. Nonetheless, to minimize angle dependencies, it is important for the pin to be directly adjacent the indicator holes (like scissor arms) rather than separated by a large distance compared to the hole diameters. Insensitivity to changes in viewing angle and sample position will result in more robust measurements and standardized results between multiple patients and clinicians. This quality also improves comparisons of bone healing with respect to time. These results indicate that this passive sensor is effective for monitoring amplified plate bending from axial loading using standard radiography techniques. Furthermore, the internal scale’s simple hole pattern is easily understood without significant training or instruction. The working principle of this sensor can potentially be translated to other applications including spine fusion and hip fracture fixation.

The FEA model demonstrates that an easily detectable indicator pin displacement of 0.5 mm corresponds to a maximum callus strain of 1.5%. This amount of strain is acceptable for a soft callus and is most likely safe for weight bearing. Some strain stimulation is necessary to induce callus formation, and strain magnitudes up to 50% of the fracture gap are tolerated in early fracture healing. In the final stages of healing soft callus can reach strains of up to 2% while still being osteoinductive^17^. The FEA model also showed that as the callus stiffens the maximum principal strain thereof decreases. This reduction in callus strain positively correlated with a decrease in pin displacement.

The proposed sensor provides physicians with an objective method to monitor fracture healing. The sensor consistently tracks interfragmentary displacement in human cadaver trials through standard radiography with approximately 250 µm resolution. Initial FEA results show the sensor is capable of measuring clinically relevant values of fracture callus strain as the bone heals. Therefore, the sensor provides an effective, non-invasive method of objectively determining when it is safe for a patient to return to weight bearing.

## Methods

### Finite element analysis

The modelling software used in this work was Ansys 15.0. The tibial computational model was based on an industry standard, mechanically equivalent model of the human tibia, provided by Pacific Research Labs (Sawbones^®^ Model #3402). Both the anatomy, cortical and cancellous geometries, and material properties were provided and are commonly used as anatomical models. The geometry includes both cortical and cancellous regions of a large tibia (42 cm in length). Material properties for cortical and cancellous bone used in the FEA are presented (Table 1).

**Table 1:** Simulated Bone Material Properties.

	Longitudinal Tensile		Transverse Tensile		Compressive	
	Strength (MPa)	Modulus (GPa)	Strength (MPa)	Modulus (GPa)	Strength (MPa)	Modulus (GPa)
Cortical	106	16	93	10	157	16.7
Cancellous	—	—	—	—	6	0.155

The table shows the material properties used in the FEA presented in this paper. These material properties are provided by Sawbones^®^ and are based on ASTM D-638 and D-695.

The model geometry was altered to include fixation blocks on both the proximal and distal ends of the tibia that are oriented to match an anatomical stance for the facilitation of loading conditions. A 10 mm fracture located on the mid-section of the tibia was also added. Orthopedic screws, plate, and sensor geometries were then positioned above the fracture.

A compressive load of 400 N (½ body weight) was applied to the block attached to the proximal end of the tibia. The distal block was defined as a fixed support with no degrees of freedom. The corresponding displacement of the indicator pin relative to the scale was then examined. An additional series of FEA models were performed with the 10 mm fracture geometry re-inserted with stiffness values ranging from 0.001–100% of longitudinal and transverse stiffness of intact bone. These models were subjected to the same loading conditions as the model with a 10 mm fracture gap and the corresponding indicator pin displacements were examined. The range of fracture stiffness is meant to simulate the mechanical capabilities of the fracture callus in load sharing with the implant as the bone heals.

### Device fabrication

**Figure 6:**
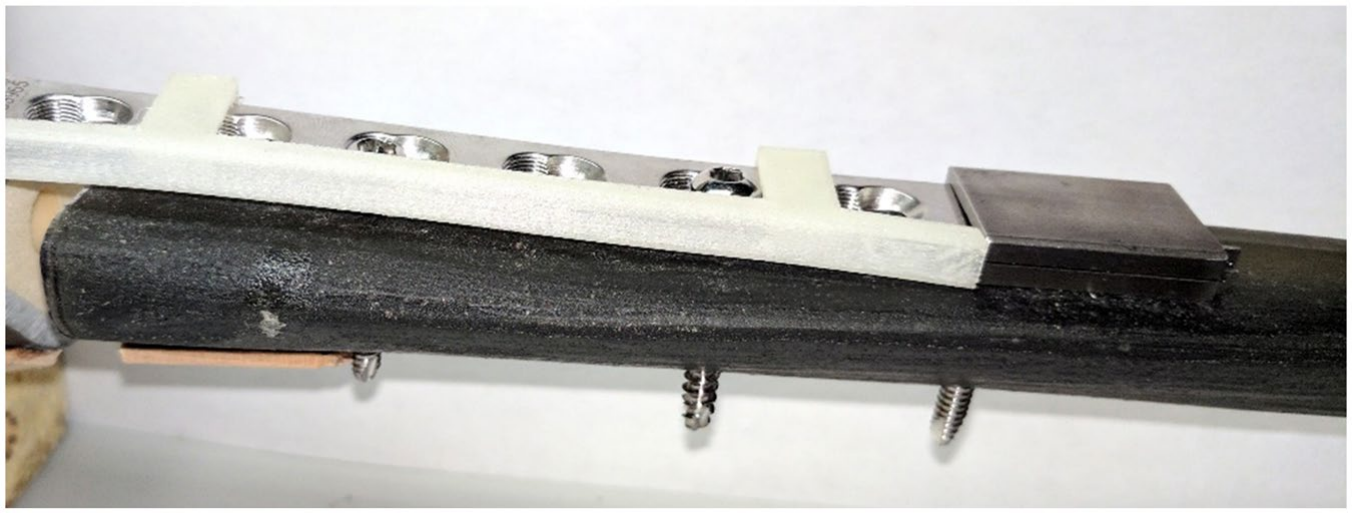
Photo of prototype pin cover.

The passive sensor device consists of two components fabricated from stainless steel and a 0.5 mm diameter tungsten indicator pin. These materials are widely used in orthopedic implants, well tolerated biologically, and robust over decades. Tungsten was chosen as a biocompatible radiopaque material with high young’s modulus, as well as relatively inexpensive. However, other materials can be used. In particular, only the tip of the indicating rod needs to be narrow and radiopaque. As long as the rod is read against soft tissue (as opposed to through a plate), other materials such as stainless steel would be appropriate. The two stainless steel components are machined to match the profile of an existing orthopedic plate. These components can be secured to the plate via clamping forces applied by a single set screw located on the sides of each component. Positioning the sensor on the top and side of the implant makes interference from bone in-growth unlikely. Interference of the pin from soft tissue in-growth is also prevented by the pin cover and can be further reduced by increasing the stiffness of the indicator pin. A prototype pin cover was fabricated out of polylactic acid (PLA) using 3D printing (Fig. 6). Although this material is biodegradable, other biocompatible materials such as polyetheretherketone (PEEK) or polydimethylsiloxane (PDMS) could also be used. For the remainder of the experiments presented here the pin cover was removed to visualize operation of the device.

One of the stainless steel components is positioned on the proximal side of the fracture and supports the cantilevered tungsten pin on the side of the orthopedic plate. The second stainless steel component is secured on the end of the orthopedic plate that is located on the distal side of the fracture. A scale consisting of five 0.5 mm diameter holes is precision machined into the second component. The holes are positioned in two columns and have an equal spacing between them of 0.5 mm in both the vertical and horizontal directions. The tungsten indicator pin is manually bent to match the contour of the plate and to align the pin tip to a starting position relative to the scale.

### Orthopedic model procedure

A Synthes tibial fracture fixation plate and a third-generation Sawbones^®﻿^ tibia model #3402 (Pacific Research Laboratories, Inc., Vashon, WA) were used to construct a fracture scenario similar to the one investigated in the FEA model. The fixation plate was implanted into the tibia and outfitted with the prototype strain sensor. A 1 cm wide fracture was created and the resulting bone piece was reinserted, allowing the bone model to act as unfractured when required. The tibia was then loaded into the Mark-10 Force Gauge and Test Stand (Mark-10 Corporation, Copiague, NY). A camera system was aligned with the gauge on the strain sensor, providing a clear view of the pin position. After photographing the sensor for “unfractured” tibia in the unloaded condition, the model was subjected to a 200 N load after which another picture was then taken. The tibia was then unloaded, and the bone piece inside the fracture was removed. The “fractured” tibia was then placed into the test setup and again photographs were taken prior to loading and after a 100 N load was applied.

### Cadaveric experiments

All methods were carried out following approval by and in accordance with the Clemson University institutional review board (IRB) as well as the relevant guidelines and regulations, including informed consent of the donor. The sensor platform was tested in a biomechanical comminuted proximal metaphyseal tibia fracture model in a human cadaver to evaluate the effectiveness of the passive sensor in indicating implant bending with radiographic readout. A Synthes 4.5 mm proximal tibia plate was surgically implanted on the tibia with a set of orthopedic screws. The indicator pin support and internal scale components were then attached to the orthopedic plate on either side of the fracture and secured with set screws. A 0.5 mm diameter tungsten indicator pin was then installed and secured with adhesive before being bent to align with the contour of the plate and internal scale.

The specimen was secured to a Mark-10 motorized tension/compression test stand (Model: ESM303) in an anatomical stance for axial compression testing. The goal of the test was to monitor the sensor response using radiography under a series of compressive loads with both intact and fractured bone. The intact human cadaver tibia was loaded in compression from 0–400 N in 100 N increments and the sensor response was recorded with radiography. An unstable fracture was then introduced to the human cadaver tibia. The fractured specimen was subjected to a series of compressive loading cycles, first from 0–200 N in 25 N increments and second from 0–100 N in 25 N increments. The sensor response was recorded with radiography at each loading step. X-ray images of the fracture site were also recorded at 0 N and 400 N loads.
